# Ceramide Profiling of Porcine Skin and Systematic Investigation of the Impact of Sorbitan Esters (SEs) on the Barrier Function of the Skin

**DOI:** 10.1021/acs.molpharmaceut.4c01245

**Published:** 2025-03-11

**Authors:** Hans Schoenfelder, Moritz Reuter, Dirk-Heinrich Evers, Michael E. Herbig, Dominique Jasmin Lunter

**Affiliations:** † Department of Pharmaceutical Technology, Faculty of Science, Eberhard Karls Universität Tübingen, Auf der Morgenstelle 8, Tuebingen 72076, Germany; ‡ RaDes GmbH, Schnackenburgallee 114, Hamburg 22525, Germany

**Keywords:** ceramides, confocal Raman spectroscopy, emulsifiers, in vitro, liquid chromatography−mass spectrometry, lipids, porcine skin, sorbitan ester, stratum corneum, trans-epidermal water loss

## Abstract

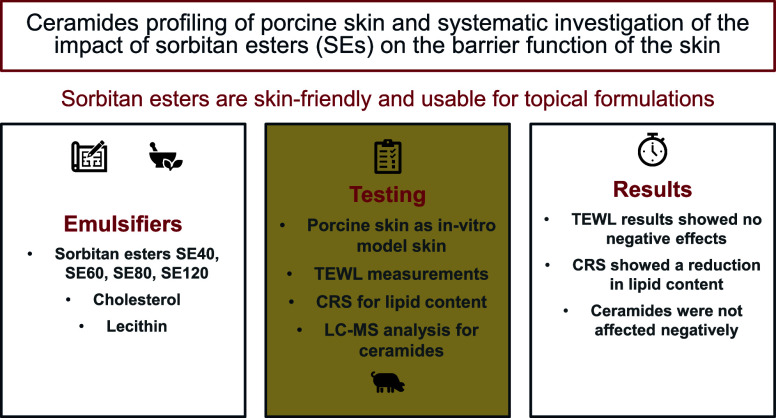

The stratum corneum (SC) lipids provide the main barrier of the skin against the environment. Ceramides make up about half of the lipids by weight and are thus of particular interest. Emulsifiers are used in a multitude of topical formulations, e.g., to stabilize emulsions against coalescence. Investigations showed that some emulsifiers have the potential to impair skin barrier function. Sorbitan esters (SEs) are frequently used emulsifiers in pharmaceutical and cosmetic dermal formulations. Further, cholesterol and lecithin were used as natural alternatives. However, information on their impact on ceramides is very scarce. Thus, we first analyzed the SEs by LC-MS with regard to their composition. Then we developed an LC-MS method to identify and quantify the ceramides in porcine skin and subsequently investigated the impact of emulsifiers on the ceramide profile. Besides the LC-MS measurements, the effect of emulsifiers on the skin barrier function was investigated by trans-epidermal water loss (TEWL) measurements and confocal Raman spectroscopy (CRS). Throughout the experiments, water was used as a negative control and sodium lauryl sulfate (SLS) as a positive control. It was found that SEs are mixtures of mono-, di-, and triesters, partially with a complex fatty acid distribution. LC-MS measurements of the total ceramide content of the SC samples revealed the SE 60 and cholesterol-treated samples to be those showing the least ceramide depletion, implying a high skin tolerability in general. The TEWL measurements showed that SEs 40, 60, 80, and 120 showed no significant changes in skin barrier function. The lipid content, measured by CRS, was mostly decreased except for SE 120. Conformation, chain order, and SC thickness, also measured by CRS, showed no significant differences. These detailed investigations lead to the view that SEs are skin-friendly substances and can be used for topical applications, e.g., those commonly used to treat skin diseases.

## 1 Introduction

**Figure 1:**
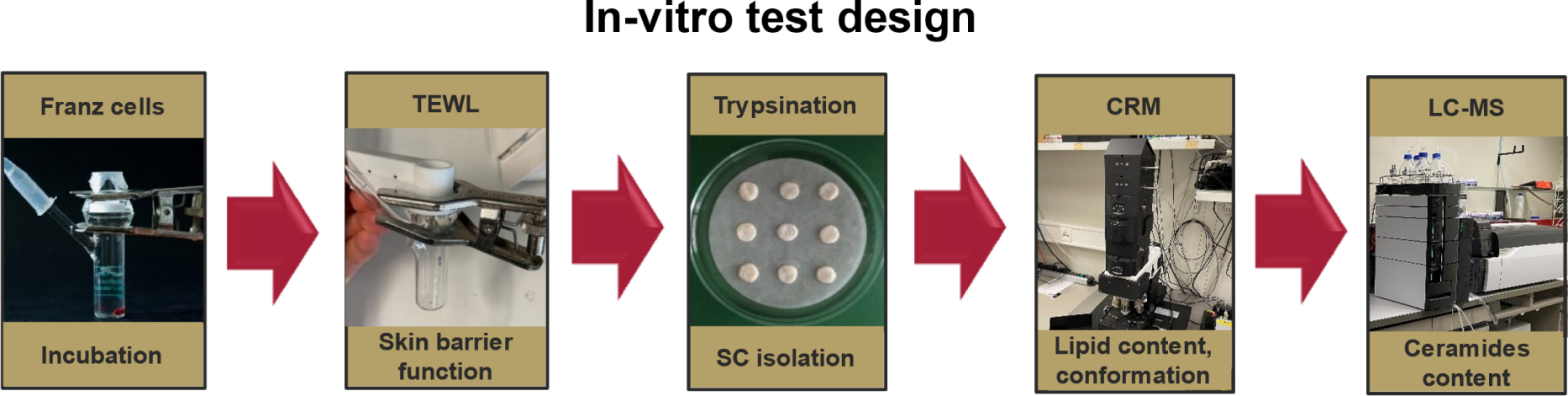
Overview of the analysis of the SC with Franz cells, TEWL measurements, trypsinization step, CRS, and LC-MS measurements.

Porcine skin is one of the most frequently used surrogates of human skin.^1,2^ It compares to human skin in terms of thickness, number of hair follicles, and immune cells.^3^ Regarding the penetration of exogenous substances, like pharmaceutical as well as cosmetic actives or excipients, porcine skin, of all animal skins, yields results closest to human skin.^4,5^ Porcine skin is also used to investigate the impact of exogenously applied substances on the skin barrier function.^6−9^ Here, for example, the impact of emulsifiers like polyethylene glycol (PEG)-ethers and polysorbates has been studied recently.^10,11^ The skin barrier function is provided mainly by SC lipids. These consist of 50% (m/m) ceramides, 25% (m/m) free fatty acids, and 25% (m/m) cholesterol and its derivatives.^12^ The ceramide portion, comprising a wide range of ceramide classes as well as chain lengths, their conformation, and packing order, has been shown to play a pivotal role in maintaining skin barrier integrity.^11,13−18^ It is known that the ratio of ceramides, free fatty acids, and cholesterol is similar in human and porcine skin. Studies exist investigating the exact composition of the SC lipids of porcine skin with high-accuracy analytic methods like LC-MS.^19,20^ These approaches with porcine skin started, for example, with Wertz and Downing in the 80s^21^ and continued with human and mice studies later.^22^ The aim of our study was to determine the ceramide profile, with the most abundant ceramide classes as well as the most prevalent chain length ranges in porcine SC, to ensure the robustness and reliability of the method of ceramide analysis in porcine skin by LC-MS and subsequently use this method to investigate the impact of emulsifiers on skin barrier integrity. To obtain a full picture, the methods of trans-epidermal water loss (TEWL) and confocal Raman spectroscopy (CRS) were also applied, as seen in Figure 1. TEWL is a parameter conventionally used in *in vivo* studies as a measure of skin barrier function and to identify the impact of cosmetic or pharmaceutical treatments on the skin barrier.^23,24^ CRS allows to investigate the total amount of lipids in a skin sample as well as the chain order of the lipids.^11,17,18,25−27^ The results of our multimodal approach were expected to give a comprehensive picture of the impact of emulsifiers on skin barrier function. SEs, cholesterol, and lecithin were chosen to be used as examples of classical chemical emulsifiers and natural alternatives. SEs are among the most common w/o-emulsifiers in the field of pharmaceutically used and cosmetic-used emulsifiers.^29−31^ They are structured as esters of sorbitan, a sorbitol derivative, and fatty acids, e.g., stearic acid for sorbitan monostearate. The various fatty acid combinations lead to a wide field of different chemical properties, like different hydrophilic–lipophilic balance (HLB) values, from 4.3 for sorbitan monooleate to 8.6 for sorbitan monopalmitate. SEs were shown, among others, by Karande et al. to enhance overcoming the skin barrier combined with sodium lauroylsarcosinate (NLS).^28^

## 2 Materials and Methods

### 2.1 Materials

SLS, sodium chloride, and potassium chloride were obtained from Caesar and Loretz GmbH (D-Hilden, Germany). LC-MS-Grade 2-propanol, methanol, acetonitrile, formic acid, acetic acid ethyl ester and, in normal quality, disodium hydrogen phosphate and potassium dihydrogen phosphate were obtained from Carl Roth GmbH & Co. KG (D-Karlsruhe, Germany). MS-grade ammonium acetate was obtained by VWR International GmbH (D-Darmstadt, Germany). *Tert*-butylmethyl ether was obtained by Supelco (Sigma-Aldrich Chemie GmbH, D-Taufkirchen, Germany). HPLC-Grade dichloromethane was obtained from Fisher Scientific U.K. Limited (UK-Loughborough/Leicestershire). SEs, including sorbitan monopalmitate (SE 40), sorbitan monostearate (SE 60), sorbitan monooleate (SE 80), and sorbitan monoisostearate (SE 120), were purchased from Croda GmbH (D-Nettetal, Germany). LC-MS-Grade ammonium formate and, in normal quality, trypsin (from porcine pancreas, lyophilized powder, type II–S) and trypsin inhibitor (from Glycine max (soybean), lyophilized powder) were obtained by Sigma-Aldrich Co. (MO-St. Louis, USA). LC-MS standards CER1 (d18:1/26:0/18:1(d9)) (*N*-[26-oleoyloxy(d9)hexacosanoyl]-d-erythro-sphingosine), deuterated ceramide lipidomix (*N*-palmitoyl(d7)-D-erythro-sphingosine, *N*-stearoyl(d7)-D-erythro-sphingosine, *N*-lignoceroyl(d7)-D-erythro-sphingosine, *N*-nervonyl(d7)-D-erythro-sphingosine), CER3(d9) (*N*-palmitoyl(d9) D-ribo-phytosphingosine), CER5–2’R(d9) (*N*-(2′-(R)-hydroxypalmitoyl(d9)) D-erythro-sphingosine), CER6–2’R(d9) (*N*-(2′-(R)-hydroxypalmitoyl(d9)) D-ribo-phytosphingosine), CER7–2’R, 6R (d9) (*N*(2′-(R)-hydroxypalmitoyl(d9)) 6R-hydroxysphingosine), CER8(d9) (*N*-palmitoyl(d9) 6R-hydroxyphingosine), CER9(d9) (t18:0/26:0/18:1(d9)) (*N*-[26-oleoyloxy(d9) hexacosanoyl]-D-ribo-phytosphingosine), CER10(d9) (*N*-palmitoyl(d9) dihydrosphingosine), and CER11–2’R(d9) (*N*-(2′-(R)-hydroxypalmitoyl(d9)) D-erythro-sphingosine) were obtained from Avanti Polar Lipids Incorporation (AL-Birmingham, USA). Parafilm was obtained from Bemis Company Inc. (WI-Oshkosh, USA). All aqueous solutions were made with ultrapure water from Elga Maxima (GB-High Wycombe, Great Britain). Phosphate-buffered saline (PBS) was prepared using sodium chloride and potassium chloride obtained from Caesar and Loretz GmbH (D-Hilden, Germany), and disodium hydrogen phosphate, and potassium dihydrogen phosphate obtained from Carl Roth GmbH & Co. KG (D-Karlsruhe, Germany). Nitrogen was obtained from an in-house tank, and argon 5.0 was delivered by Westfalen AG (D-Muenster, Germany). Porcine ear skins (German landrace; age: 15–30 weeks; weight: 40–64 kg) were provided by a local butcher. The Department of Pharmaceutical Technology at the University of Tuebingen has been registered for the use of animal products (registration number: DE 08 416 1052 21).

### 2.2 LC-MS Analysis of the Composition of SEs

SEs were analyzed by an ultrahigh-performance liquid chromatography (UPLC) system (ACQUITY UPLC H-Class PLUS, Waters) connected to a single quadrupole mass spectrometer (QDa, Waters, mass range 30 to 1250 *m*/*z*) as a detector. An Acquity UPLC HSS Cyano, 100 Å, 1.8 μm, 2.1 mm × 50 mm UPLC column from Waters GmbH (D-Eschborn, Germany) was used. Prior to analysis, SEs were dissolved at a concentration of 0.5 mg/mL in methanol. The column temperature was set to 45 °C and the autosampler temperature to 25 °C. The injection volume was set to 1 μL, and the flow rate of the eluent was 0.5 mL/min. In total, three mobile phases were used: A: 10 mM ammonium acetate and 5 mM acetic acid, B: acetonitrile +10% *tert*-butylmethyl ether, and C: methanol. Each run was conducted by using individual linear gradients. The detection was carried out with the respective 1-fold charged ammonium ions at a cone voltage of 15 V.

### 2.3 Preparation of Emulsifier Solutions/Dispersions

Testing solutions/dispersion of SE 40, SE 60, SE 80, SE 120, lecithin, cholesterol, and SLS were prepared as 1% solution/dispersion (w/w) in water, sonicated (Bandelin Sonorex, Bandelin electronic GmbH & Co. KG, D-Berlin, Germany) for 15 min, and vortexed for 1 min (IKA Vortex 2, IKA-Werke GmbH & Co. KG, D-Staufen, Germany) as described previously.^24^

### 2.4 Porcine Ear Skin Preparation

Porcine skin is histologically and morphologically comparable to human skin; therefore, it was chosen as a surrogate for human skin.^1,2^ The skin was prepared as described in earlier publications of our group.^32,33^ Fresh pig ears were cleaned with isotonic saline. Full-thickness skin was removed from the cartilage, and blood was removed with isotonic saline and cotton swabs. The obtained postauricular skin was dried with soft tissue. The skin was sliced into strips of about 3 cm in width. The skin was stretched onto a Styrofoam plate to reduce the impact of wrinkles. With an electric hair trimmer (QC5115/15 Philips Electronics, NL-Eindhoven, Netherlands), bristles were cut to about 0.5 mm in length. After being dermatomed to a thickness of 1.0 mm (Dermatom GA 630 Acculan 3 TI Aesculap AG and Co. KG, D-Tuttlingen, Germany), the skin was punched out into circles of 25 mm diameter and placed in the freezer at minus 28 °C wrapped in aluminum foil. On the day of the experiment, the samples were thawed to room temperature on a piece of paper tissue soaked with phosphate-buffered saline pH 7.4 (PBS).^33^

### 2.5 Incubation of Skin Samples in Franz Diffusion Cells

Franz diffusion cells are a typical type of analytical setup for determining skin absorption *ex vivo* and the method described herein has been used extensively by our group in the past as described in previous publications.^11,17,32,33^ Prior articles from our group provide a thorough discussion of the strategy for the incubation of skin samples with emulsifiers.^11,34^ Franz diffusion cells (Gauer Glas, D-Püttlingen, Germany) were filled with 12 mL of degassed, prewarmed (32 °C) PBS as the receptor fluid. The skin samples were placed on top of the acceptor compartments, and the donor compartments were placed on top of the skin. The Franz diffusion cells were placed in a water bath at 32 °C (Lauda type Alpha, Lauda Dr. R. Wobser GmbH & Co. KG, D-Lauda-Königshofen, Germany). The receptor fluid was continuously stirred at a 500-rpm rate (Variomag Poly 15, Thermo-Scientific, Thermo Electron LED GmbH, D-Langenselbold, Germany). After an expeditious equilibration period of 30 min, the initial TEWL values were generated with the protocol described in section 2.6. After the initial TEWL measurements, 1 mL of each emulsifier solution/dispersion, water (negative control), or SLS (positive control) was applied to the respective skin samples. Each donor compartment was then covered with a piece of parafilm to reduce evaporation. After a 4-h incubation, the residual formulation was wiped off the skin, and the second TEWL measurement was performed.^11,34^ Experiments were performed in triplicate.

### 2.6 Measurement of Trans-Epidermal Water Loss (TEWL)

The TEWL was measured using the basic device Multi Probe Adapter MPA 6 and the probe In-vitro-Tewameter VT 310 (Courage & Khazaka electronic GmbH, D-Köln, Germany), and calculated by the respective software. The room temperature was 22 °C and the relative humidity (RH) was 25% (Klima logg pro TFA 30.3039 IT; Dostmann GmbH & Co. KG, D-Wertheim, Germany). After 30 min, each Franz diffusion cell was taken out of the water bath, and 2 mL of PBS was taken out of the Franz diffusion cell acceptor compartment with a needle attached to a 2-mL syringe. After taking off the donor compartment, the skin was dried with tissues and cotton swabs. The probe was put on the acceptor compartment, and the initial TEWL value was measured. Then, the measurement started with a measurement time of 90 s for each run. A minimum of five measurements were taken, which were regarded as the equilibration phase. More than five measurements were deemed necessary if the difference between three subsequent measurements exceeded ±1.00 g·m^–2^·h^–1^. After the last measurement, the probe was taken off, and the donor chamber was put back on top of the Franz diffusion cell. The withdrawn 2 mL of PBS was refilled , and the respective emulsifier solution/dispersion was applied to the skin, and starting the 4 h incubation. Afterward, the emulsifier solution/dispersion was discarded, the skin was dried, and the TEWL was measured as described above. The change in TEWL is the margin of the TEWL before and after the 4 h of incubation in g·m^–2^·h^–1^. This procedure was validated and is already described in detail in a prior article by our group.^24^

### 2.7 SC Isolation and Drying

The SC was isolated by a trypsin digestion process as described by Kligman.^17,35^ This isolation procedure has been proven not to influence the lipid content or lipid lamellar organization. The obtained skin samples were placed dermal side down on filter paper soaked with 0.2% trypsin diluted in PBS solution. After the incubation of skin samples overnight, the digested SC was peeled off gently and immersed in 0.05% trypsin inhibitor diluted in PBS solution for 1 min. Afterward, the isolated SC was washed with fresh purified water five times. Before the measurements, samples were stored in a desiccator for drying for 3 days.^36^

### 2.8 SC Thickness Measured by Micrometer Gauge

After treatment with different emulsifiers, the SC thicknesses were measured with the eddy current method using a Fischer DUALSCOPE FMP20 portable instrument equipped with an FTA3.3–5.6H probe (Helmut Fischer, D-Sindelfingen, Germany). To reduce the effects of hair, which can accidentally cause errors when measuring the distance between the SC surface and sample substrate, hair was very gently removed when washing the isolated SC in water. SC samples were then put on round glass plates for DUALSCOPE and the following CRS measurements. Each skin sample was measured over 12 times at different places. The averages and standard deviations were then calculated for comparison.^36^

### 2.9 CRS Measurements

#### 2.9.1 CRS Setup

After drying, the SC sheets were fixed onto the scan table of the alpha300 R confocal Raman microscope (WITec GmbH, D-Ulm, Germany). This CRS device was equipped with a 532-nm excitation laser, a UHTS 300 spectrometer, and a DV401A-BV CCD camera. To avoid skin samples’ damage due to high laser intensity, the laser power was set to 10 mW, adjusted by the optimal power meter (PM100D, Thorlabs GmbH, D-Dachau, Germany). The objective 100 × 0.9 NA (EC Epiplan-neofluar, Carl Zeiss, D-Jena, Germany) was used. During the measurement, the light was focused through the objective onto the SC surface. The backscattered light from the SC was then dispersed by an optical grating (600 g/mm to obtain the spectral range from 0–4000 cm^–1^ or 1800 g/mm to achieve higher spectral resolution for analysis of trans–gauche-ratio). The scattered light was collected and analyzed on a charge-coupled device (DV401A-BV CCD detector) which had been cooled to −60 °C in advance. The CRS measurements were performed based on a method developed by Zhang et al.^27^

The spectra were collected with an integration time of 5 s and 5 accumulations. To achieve spectral signals of lipids from the skin surface and measure SC thickness at the same time, the spectra were detected with the focus point moving from −15 μm beneath the skin to 15 μm above the skin. The spectra were recorded with a step size of 1 μm. The skin surface was determined as the half-maximum of the keratin signal intensity (ν (CH_3_), 2920–2960 cm^–1^) as described before.^27,34,36,37^

#### 2.9.2 Preprocessing of CRS Spectra

The obtained Raman spectra were edited with spectral cosmic ray removal (CRR), followed by a principal component analysis (PCA) and background subtraction (SubBG), which was performed by WITec Project 6.0 Software (WITec GmbH, D-Ulm, Germany). The background subtraction in the HWN region was applied with the mode shape 300, and the fingerprint region and transgauche-ratio were applied with the mode polynomial third order and zero smoothing points. After that, the AUC extracted in this study was the integrated area under a specified peak of the spectrum and could be calculated using the trapezoidal method on WITec Project 6.0 Software.^11,34^

#### 2.9.3 Skin Lipid Content Analysis by CRS

Based on previous research, the spectral signal in the fingerprint region is more sensitive to analyze the skin lipid content than that in the high wavenumbers region.^34^ In detail, the δ (CH_2_, CH_3_)-mode at 1425–1490 cm^–1^ is derived to a large extent from lipids in the SC. The ν (C=O)-mode at 1630–1710 cm^–1^ (amide I mode) is derived from proteins. In general, the spectral intensities vary to some extent between different skin samples and different donors. The amide-I mode displays the least variation within one donor or among different donors.^25,38^ To account for the described variation of spectral intensity, the lipid-related signals were normalized to the amide-I signal for calculating the total lipid content.^34,36^

#### 2.9.4 Analysis of Lipid Conformation by CRS

The lipid conformation was determined by the trans–gauche ratio in the fingerprint region. At the positions of 1060 and 1130 cm^–1^ the trans conformation is displayed. The peak at 1080 cm^–1^ represents the gauche conformation. Gauche conformation represents a more disordered state of lipids, whereas lipids in trans conformation are more ordered.^26,38^ The AUCs under the respective peaks are used to calculate the trans–gauche–trans ratio as AUC_1080_/(AUC_1060_ + AUC_1130_) and was described in detail by Snyder et al.^39^ Accordingly, a higher value represents a more disordered conformation of the lipids. This procedure was also described in detail by our group.^11,27^

### 2.10 LC-MS Analysis of Ceramides

#### 2.10.1 Sample Preparation for LC-MS Ceramide Analysis

The glass slides carrying the SC sheets were crushed and transferred into 2 mL reaction vessels (Eppendorf SE, D-Hamburg, Germany). The vessels were then filled with 2 mL of the extraction medium, containing volume of eight parts methanol to two parts ethyl acetate, as well as the internal standards ceramides NS16d7, NS24d7, as well as Ceramide EOS26d9. The mixture was then shaken for 3 h (IKA Vibrax VXR basic, IKA-Werke GmbH & Co. KG, D-Staufen, Germany), sonicated for 15 min (Bandelin Sonorex, Bandelin electronic GmbH & Co. KG, D-Berlin, Germany), and vortexed for 1 min (IKA Vortex 2, IKA-Werke GmbH & Co. KG, D-Staufen, Germany). The sample tubes were then centrifuged at 13,400 rpm for 10 min (MiniSpin, Eppendorf SE, D-Hamburg, Germany), and the supernatant was taken off and filtered into HPLC vials using a 0.2-μm PTFE filter (Chromafil, Macherey-Nagel GmbH & Co. KG, D-Düren, Germany).

#### 2.10.2 LC-MS Measurements of Ceramides

Samples were analyzed using reversed-phase UPLC (UPLC, Nexera LC-40, Shimadzu Corporation, Kyoto, Japan) coupled with mass spectrometry (LCMS-8045, Shimadzu Corporation, Kyoto, Japan). The UPLC separation step was performed using a binary gradient, with the total method duration being 20 min. The UPLC used a flow rate of 0.4 mL/min and the following gradient: eluent A: 10 μM ammonium formate in water and eluent B: 0.1% (v/v) formic acid in isopropanol/acetonitrile 50/50. Concentration of eluent B over time: 0 min: 10%; 0.5 min: 20%; 1 min: 40%; 12.5 min: 92.5% (nonlinear curve); 12.6 min: 100%; 17 min: 100%; 18 min: 20%; and 20 min: 20% (flushing step). Ceramide content was determined in the mass spectrometer by using the following settings: ESI positive ionization mode; interface voltage: 3 kV; interface temperature: 220 °C; and desolvation temperature: 355 °C. Ceramides were quantified using multiple reaction monitoring (MRM) to obtain higher specificity in the extraction matrix: precursor ions in the Q1 were either the [M + H]^+^ ion for the ceramide groups NP, AP, NDS, ADS, EOS, EOP, and EODS, or [M – H_2_O + H]^+^ for ceramide groups NS and AS. After fragmentation, the LCB-specific fragments^22^ were used for product ion quantification of the ceramides. A full table of the precursor ions, collision energies, and corresponding product ions can be found in the Supporting Information in Table S3. The measured ceramide species comprise a chain length range of C16–C28 for non-EO ceramides as well as C28–C35 for EO ceramides. To account for variability in ionization, the obtained intensities were then normalized by the internal standards of Ceramide NS16d7, NS24d7, as well as Ceramide EOS26d9, depending on the retention time as well as the ceramide species; see Supporting Information for details. Concentrations were then calculated from the normalized intensities using calibration curves of ADS16d9, AP16d9, NP16d9, AS16d9, NS16d9, NDS16d9, as well as EOS26d9 and EOP26d9. For the EODS ceramides, the ADS16d9 calibration curve was used for calculation of the concentrations. The water-treated sample (negative control) was used for the ceramide profiling. The calculated concentrations were then normalized using the mass of the extracted SC to obtain the final ceramide content. Raw data processing was performed using Shimadzu LabSolutions, while subsequent and standard calculations were performed using Microsoft Excel as well as R.

### 2.11 Statistical Analysis

The statistical analysis is split into two parts: the data sets from the CRS and thickness measurements constitute 5 discrete measurements for each of the three skin samples, resulting in *n* = 15 observations for every emulsifier treatment and the water reference. These data sets were then analyzed using a Kruskal–Wallis test with a posthoc Dunn’s test investigating differences between the emulsifier treatments and the water reference. Nonparametric tests were used as the data distribution of these measurements failed to show normality. The box plots represent all measurements, the points are outliers. For the TEWL, points are measured samples, and for ceramide content measurements, one observation was derived for each of the three skin samples used for analysis, resulting in *n* = 3 for every emulsifier treatment and the water reference. The data obtained from these measurements were analyzed using an analysis of variance followed by a posthoc Fisher’s Least Significant Difference test, comparing the effect of each emulsifier treatment to the water reference. The statistical processing was carried out in GraphPad Prism 8.0 (GraphPad Software Inc., La Jolla, CA, USA). Different numbers of asterisks are used to indicate significant differences: *p < 0.05, **p < 0.01, and ***p < 0.001.

## 3 Results

### 3.1 Results on Composition of SEs

LC-MS analysis revealed that all SE variants investigated are a mixture of mono-, di-, and triesters of sorbitan with fatty acids. The ratio of the peak areas of mono- to di- to triesters is around 1.2:3.0:1.6 in all cases, with 19.1–25.9% monoesters, 49.8–54.4% diesters, and 24.3–29.6% triesters (Table 1). When the theoretical saponification values for the distribution pattern obtained from peak area distribution are calculated, the values are 3–17% higher than the measured saponification values according to the certificates of analysis.

**Table 1:** Distribution for Mono-, Di-, and Triesters of Different Span Variants (Peak Area %)

Emulsifier name	Nominal composition	Sum of monoesters	Sum of diesters	Sum of triesters
SE 40	Sorbitan monopalmitate	19.1	51.3	29.6
SE 60	Sorbitan monostearate	19.7	54.4	25.9
SE 80	Sorbitan monooleate	20.0	52.0	28.0
SE 120	Sorbitan monoisostearate	25.9	49.8	24.3

The theoretical saponification values were calculated for all batches of the different SE variants (based on the fatty acid composition, assuming that these are exclusively sorbitan mono variants). The calculated values are all below actual values found and below the lower specification limit (Table S1).

**Figure 2:**
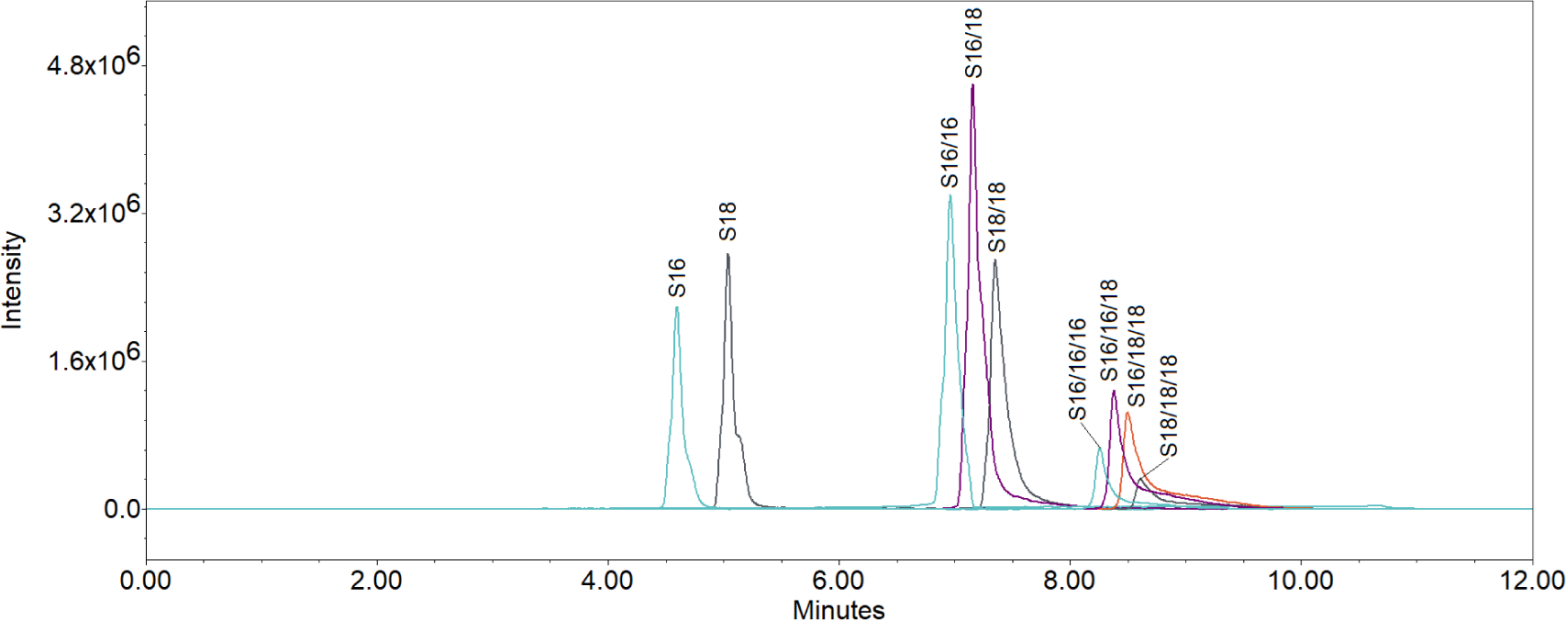
Spectrum of SE 60 with intensity over time: first S16 and S18 as monoesters, second S16/16, S16/18, and S18/18 as diesters, and third S16/16/16, S16/16/18, S16/18/18, and S18/18/18 as triesters.

This confirms that a relevant fraction of di- and triesters must be present, but the values for these species calculated from the peak areas may be too high. Potential explanations for this discrepancy could be the presence of certain amounts of unesterified compounds (free sorbitan and anhydrides of sorbitan and/or free fatty acids) or different ionization behavior of the mono-, di-, and triesters in the mass spectrometric analysis. In future studies, further refinement of the analytical methods is needed to obtain a precise understanding of the excipient’s composition with regard to the ratio of mono-, di-, and triesters. With the exception of SE 40, for which only palmitic acid could be determined in the relevant concentration, all other SE emulsifiers contain more than one fatty acid species in relevant concentrations. In Figure 2 the composition of SE 60 (designation: sorbitan monostearate) is shown. It shows the similar amounts of palmitic (C16) and stearic (C18) acid. The absolute content of sorbitan monopalmitate (S16) and sorbitan monostearate (S18) is around 10% (Area %) for both. With regard to their peak area distribution, the S16_16 and the S18_18 diesters are both contained at around 15%, and the mixed diester S16_18 at around 23%. The uniformly composed triesters S16_16_16 and S18_18_18 are present at around 3%, whereas the two mixed triesters S16_16_18 and S16_18_18 are present at around 9%. The composition is in agreement with that of the Pharm. Eur. monograph “sorbitan stearate”, which requires 40.0% to 60.0% of stearic acid and a sum of the contents of palmitic and stearic acids: minimum 90.0% for sorbitan stearate (type I).^40^ Whereas Pharm. Eur. provides specifications of the fatty acid distribution for sorbitan stearate, sorbitan palmitate, and sorbitan oleate, no specifications of the ratios of mono-, di-, and triesters are given.

These findings provide important evidence for a better understanding of SE emulsifiers. They can all be considered a mixture of species of different polarity and HLB values. SE 60 contains species with an HLB of 2.1 (sorbitan tristearate) to 6.7 (sorbitan monopalmitate)^41^ and can, therefore, be considered a lipophilic complex emulsifier.

### 3.2 TEWL Results

**Figure 3:**
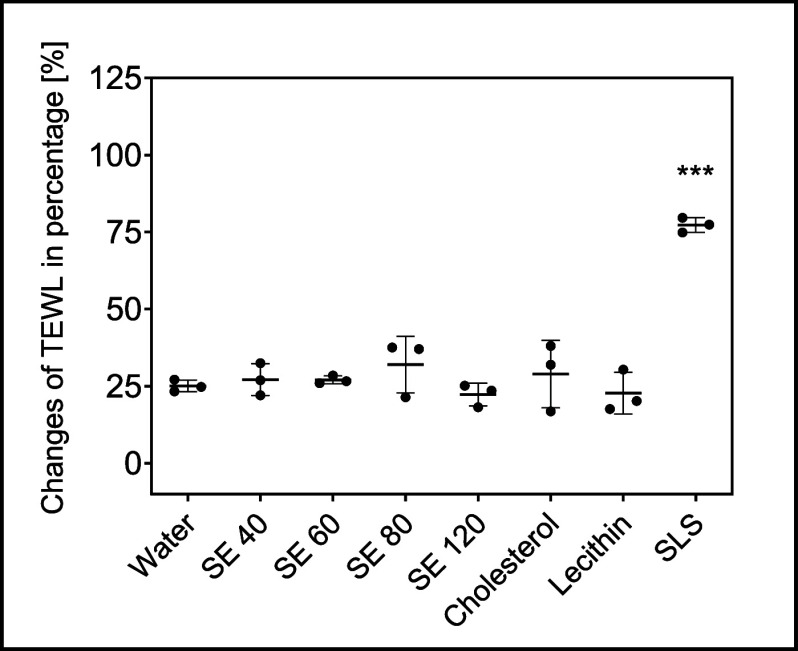
TEWL measurements after 4 h of incubation with 1% aqueous solutions/dispersions of emulsifiers with water as negative control and SLS as positive control. Results are shown as the change of TEWL in percentage. Emulsifiers SE 40, SE 60, SE 80, SE 120, cholesterol, and lecithin are in between, in which only SLS showed significant negative effects. Mean ± SD, *n* ≥ 3. *p < 0.05, **p < 0.01, ***p < 0.001.

The TEWL is not just a value to describe skin barrier function; it is also a kind of quality control for the utilized skin samples in ex vivo experiments. According to the EMA draft guideline on quality and equivalence of topical products^42^ before any experiment, one should check for skin damage or other parameters that might influence the results. We investigated the TEWL change after incubation of skin samples with emulsifier solutions/dispersions to elucidate their impact on skin barrier function and possible damage thereof. Figure 3 shows the results of the TEWL measurements. As expected, SLS, as the positive control, showed significantly increased TEWL values compared to the water-treated sample used as a negative control (23 vs 77 g m^–2^·h^–1^). SLS is well-known for its skin disruptive effects^43^ and has already showed strongly increased TEWL values in previous experiments.^24^ The TEWL change as a result of treatment with SEs, cholesterol, and lecithin was not significant compared to that of the water-treated sample. Our previous research had shown that many o/w-emulsifiers impair the skin barrier *ex vivo*. Also, some w/o emulsifiers (e.g., glycerol monostearate) were found to impair the skin barrier function, while most did not (e.g., PEG-2-fatty alcohol ethers, and cetostearyl alcohol). The current results confirm the expectation that w/o-emulsifiers are not as prone to affect the skin barrier function as o/w-emulsifiers are.^11,17,18,24,44,45^ Lecithin was also shown by other research^46,47^ to be a mild emulsifier, which was again confirmed herein.

### 3.3 CRS Results

**Figure 4:**
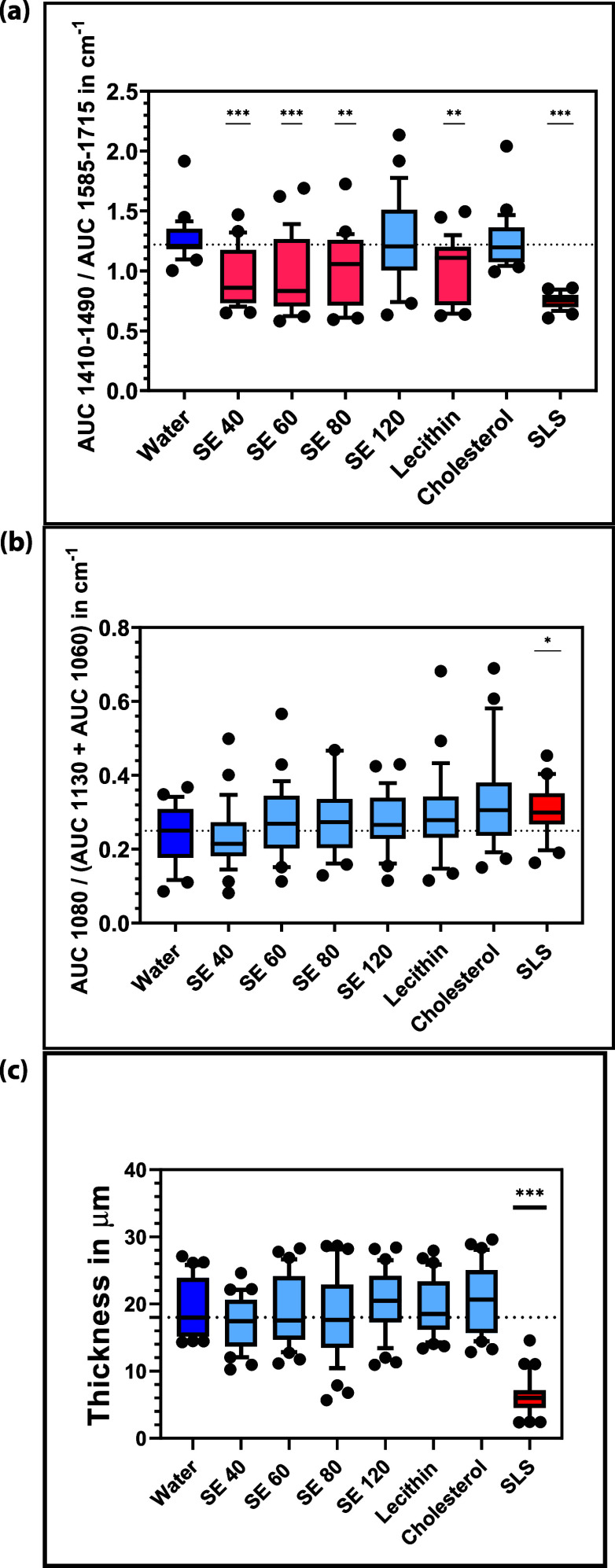
(a) CRS fingerprint lipid content measurements after 4 h of incubation with 1% aqueous solutions/dispersions of emulsifiers with water as negative control and SLS as positive control. Emulsifiers SE 40, SE 60, SE 80, SE 120, cholesterol, and lecithin are in between, in which all emulsifiers except SE 120 and cholesterol showed significant negative effects. Mean ± SD, *n* ≥ 3. *p < 0.05, **p < 0.01, ***p < 0.001. (b) CRS gauche–trans ratio measurements after 4 h of incubation with 1% aqueous solutions/dispersions of emulsifiers with water as negative control and SLS as positive control. Emulsifiers SE 40, SE 60, SE 80, SE 120, cholesterol, and lecithin are in between, in which only SLS showed a significant negative effect by a higher ratio value. Mean ± SD, *n* ≥ 3. * *p* < 0.05, ** *p* < 0.01, *** *p* < 0.001. (c) DUALSCOPE FMP20 SC thickness measurements after 4 h incubation with 1% aqueous solutions/dispersions of emulsifiers with water as a negative control and SLS as a positive control. Emulsifiers SE 40, SE 60, SE 80, SE 120, cholesterol, and lecithin are in between, in which only SLS showed significant negative effects. Mean ± SD, *n* ≥ 3. *p < 0.05, **p < 0.01, ***p < 0.001.

The lipid content was determined in the fingerprint region by lipid signals normalized to the amide-I-mode. Our antecedent research described this method in detail.^11,17,34^ In Figure 4a the results are shown. SLS was, as the positive control, the most different from the negative control (water) (***p < 0.001), followed by SE 40 and SE 60. Also significantly decreased were the lipid content of skin samples treated with SE 80 and lecithin. SE 120 and cholesterol did not significantly decrease the lipid content and behaved like the negative control.^11^ It should also be noted that results from emulsifier-treated samples gave higher variability than samples treated with the positive or negative control. This is an effect that has already shown up in previous research and seems to be linked to partial extraction of the lipids.^11,17,18,45^

With respect to the lipids, increased values of the trans–gauche ratio show a conformational change, which leads to lower resistance of the SC against xenobiotics.^26,38^ The results of the lipid conformation analysis are shown in Figure 4b. Here, SLS significantly increased the trans–gauche ratio and thus the disorder of lipids. All tested emulsifiers did not yield significantly different results compared to the negative control. Interestingly, SE 80 and cholesterol-treated skin samples gave higher variability than other emulsifier-treated samples. This is an interesting finding, as previous research had shown that emulsifiers that extracted lipids from the SC also induced a higher degree of disorder, as reflected by an increased trans–gauche ratio. A possible explanation may be the low degree of lipid extraction by the currently investigated emulsifiers compared to those previously investigated. Lipid extraction may be too low to result in significantly impaired lipid conformation.^11,17^

The thickness of the SC is another indicator of the effect of an emulsifier on SC integrity. After treatment with emulsifiers, the SC can become thinner, which leads to impaired skin barrier function. Figure 4c shows that the thickness of SLS-treated SC was significantly decreased by 13 μm. The other emulsifiers did not affect the SC thickness significantly. This complies with the previous measurements, which also showed no significant impact of the investigated emulsifiers on SC lipid content and conformation.

### 3.4 LC-MS Results

**Figure 5:**
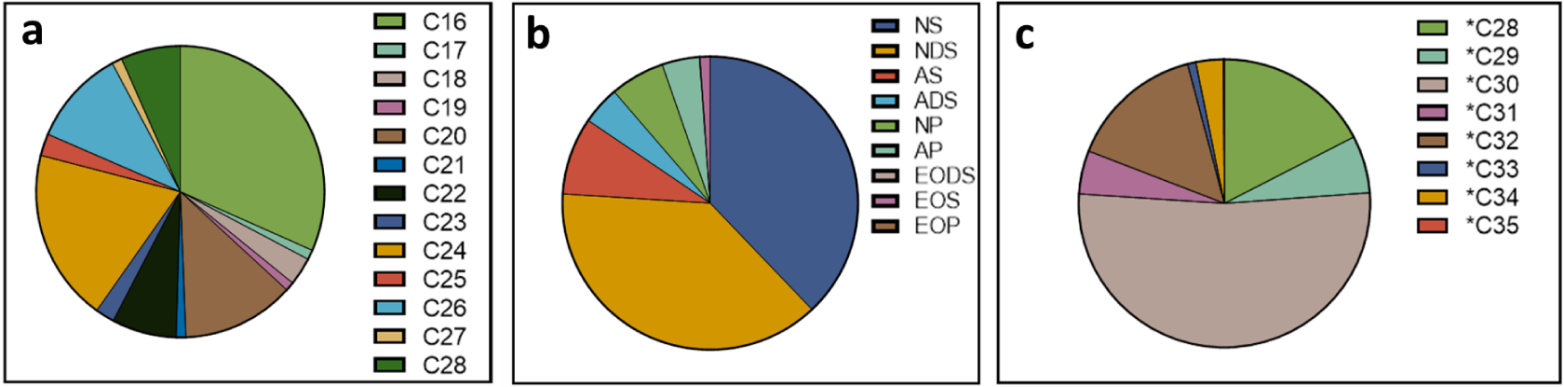
(a–c) Pie charts of the relative distribution of ceramide classes (Figure 5a), chain lengths of regular ceramides (Figure 5b), as well as the ω-esterified ceramides (Figure 5c). Detailed percentages, including the standard deviation, can be found in Table S2.

The analysis of the skin’s ceramide content yielded a clear relative lipid class and chain length distribution, as seen in Figure 5a–c. The lipophilic ceramide classes NS as well as NDS were found to be the most abundant ceramide classes present in porcine skin, while the more hydrophilic ceramide classes NP and AP were only present in low quantities, contrasting sharply with the human skin ceramide composition, where the hydrophilic ceramide classes containing phytosphingosine (overwhelmingly represented as ceramide class NP and AP) as well as hydroxysphingosine (overwhelmingly represented as ceramide class NH and AH) represent the most abundant ceramide classes.^20,22,48^ Of the ω-esterified ultralong chain ceramide classes, the sphingosine-containing EOS was the most abundant, although the detected quantity of EO-type ceramides in general was very low. Regarding the chain length distribution, the most common chain length of the regular ceramide classes is 16 carbon atoms, followed by 24 and 26 carbon atoms, respectively. Again, this contrasts with the composition generally given for human skin, where C24 and C26 can be found as the most common chain lengths. For EO-type ceramides, the by far most abundant ceramide chain length, comprising more than half of the measured ω-esterified ceramides, contains 30 carbon atoms, which mirrors the composition of human skin in this regard.^20,22,48^ Consequently, the composition of the ultralong chain ceramides of the porcine skin, in ceramide class as well as chain length distribution, shows much less difference to the human ultralong chain ceramide composition than the shorter chain length, nonesterified ceramides present in the skin.

**Figure 6:**
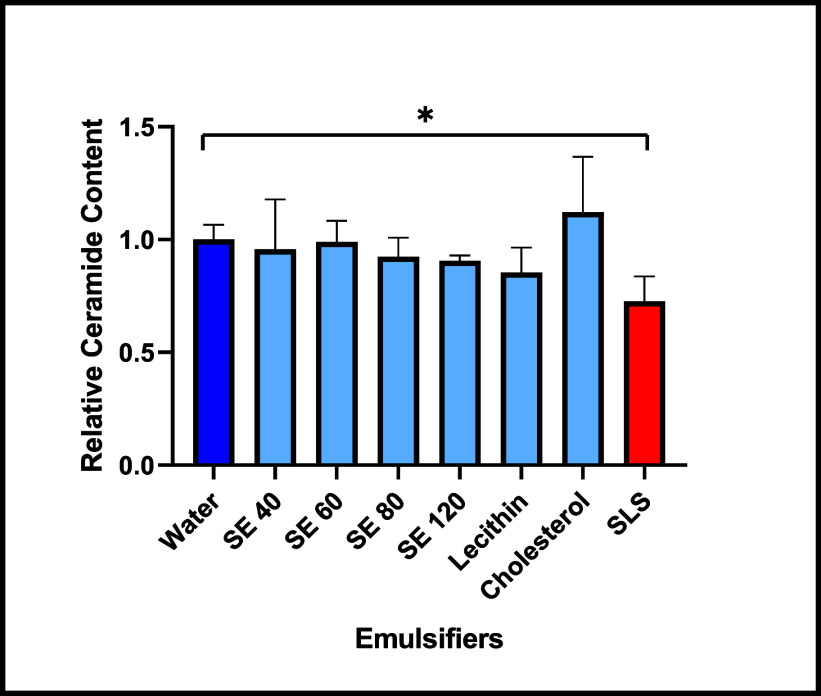
LC-MS measurements after 4 h incubation with 1% aqueous solutions/dispersions of emulsifiers with water as negative control and SLS as a positive control, showing the relative ceramide content. Emulsifiers SE 40, SE 60, SE 80, SE 120, cholesterol, and lecithin are in between, in which SLS showed significant negative effects. Mean ± SD, *n* = 3. *p < 0.05, **p < 0.01, ***p < 0.001.

The total ceramide content of the treated skin samples obtained from the LC-MS measurements is shown in Figure 6. The ceramide content is given as the relative proportion of ceramides detected per mass of the SC of the water-treated sample. The only treatment showing a significant reduction in total ceramide content compared to the water-treated control is the positive control, SLS. All other investigated emulsifiers showed no significant difference, confirming that no ceramides were extracted from the SC.

## 4 Discussion

SEs are commonly used as w/o-emulsifiers. In our current research, it was found that the investigated SEs were all mixtures of mono-, di-, and triesters with an approximate ratio of 1.2:3.0:1.6 with regard to their peak area in the chromatogram. This is in contrast to their common designation as “sorbitan monoesters” and to the HLB values assigned to them, which refer to the monoester,^41^ and demonstrates that all tested SEs consist of species of varying solubility properties and polarity, including the nonpolar triesters with HLB values around 2.^49^

While in the USP the denomination “monoesters” is still used,^50^ the current English version 11 of Pharm. Eur. states only “sorbitan esters”.^40^ Monographs are available for sorbitan stearate and sorbitan palmitate, which give no information or specification on the ratio between mono-, di-, and triesters. Therefore, it cannot be excluded that the distribution patterns of the different esters vary between suppliers. Whereas the monoesters function primarily as w/o emulsifiers, the triesters may mix with oil phases of emulsion-type formulations. The less polar di- and triesters may also contribute to favorable skin tolerability of this class of emulsifiers.

In the main part of this study, the SEs, together with the physiological emulsifiers of lecithin and cholesterol, were analyzed by a multimodal approach to achieve thorough characterization of the impact of these emulsifiers on porcine skin. This multimodal approach (TEWL, lipid order, and ceramide content) further enabled, for the first time, a comparison of results derived from different methods like TEWL, CRS, and LC-MS without bias due to the use of different samples for each method. Ohnari et al. showed another approach with human skin and the tape stripping method using TEWL, FTIR, and LC-MS, but without using the same sample for every method.^51^ Additionally, only parts of the SC were sampled by tape stripping, whereas we used the whole SC. Looking at the TEWL results, the SEs proved to be skin-friendly, causing no skin barrier impairment. In this respect, they behaved similarly to lecithin and cholesterol, which also showed high skin tolerability in the TEWL measurements. Yet, CRS measurements showed emulsifier-treated skin sites having significantly decreased lipid contents (except for SE 120 and cholesterol due to high variability), while the lipid conformation was not affected. This conformation is an indicator of the chain order of lipids: lipids in orthorhombic order are more resistant to the intrusion of xenobiotics and thus provide a higher skin barrier function.^52,53^ The results from the TEWL measurements as well as the CRS imply a mixed effect on the skin: while most of the tested emulsifiers decreased the lipid content, they did not cause lipid conformation disordering, which correlates well with the TEWL results, implying an unimpaired skin barrier function of the treated sites. The information about the lipid conformation state obtained from CRS may therefore be of higher significance for investigating possible skin barrier impairment than the total lipid content measured: as stated before, disordering of the skin lipid conformation is associated with a steep increase in skin barrier permeability. Interestingly, the penetration-enhancing effect of the disordering of skin lipids is even pronounced in certain nonionic emulsifiers, such as Polysorbate 80, which show no lipid depletion in CRS studies.^45,54^ The absence of lipid disordering effects of the investigated nonionic emulsifiers in this study demonstrates the skin tolerability of these substances, which is further substantiated by the thickness of the SC remaining constant after incubation with them. Adding to these results, the total ceramide content measured by LC-MS analysis showed the total ceramide content to not be significantly decreased after treatment with any of the investigated emulsifiers. Only SLS, as a positive control, led to a statistically significant depletion of ceramides, as is typical for this surfactant and seen in previous studies.^55,56^ For the investigated emulsifiers, these results hint toward a disconnection between the lipid content determined by CRS and the ceramide content determined by LC-MS. This could possibly be explained by depletion in fatty acids and cholesterol rather than ceramides,^57^ which will be the subject of further studies. Nonetheless, the neutral effect of the investigated emulsifiers on the ceramide content compared to the aggressive positive control further qualifies the investigated emulsifiers as skin-friendly and suitable for use in dermal products.

## 5 Conclusion

Emulsifiers that are used in topical formulations must be examined for their safe use. We found that all SEs investigated in this study, contrary to what their designations suggest, are mixtures of mono-, di-, and triglycerides. Therefore, these compounds are mixtures of species of different polarity, which may have different functions in formulations. With the combination of TEWL, CRS, and LC-MS, a multimodal analysis was performed to describe their effects on the skin. This study highlights the efficacy and usefulness of multimodal SC analysis by utilizing the synergies offered by the combination of TEWL, CRS, and LC-MS analysis. The combination of three completely different methods proved to be a versatile approach in the characterization of an emulsifier’s impact on the skin barrier function. This will facilitate the characterization of the effect of emulsifiers or other excipients of topical formulations in the future. Furthermore, these detailed investigations led to the finding that SEs, as w/o-emulsifiers, are skin-friendly substances and can be used for dermal formulations, with a low skin barrier-impairing potential comparable to that of lecithin and cholesterol, physiologically occurring substances that are known to be mild emulsifiers. As pegylated emulsifiers, in particular, have come increasingly under fire amidst health and sustainability concerns in the general public, the SEs present a PEG-free and skin-friendly alternative. The multimodal approach to skin barrier impact characterization can easily be adapted to in vivo analysis, requiring only minor modifications to the LC-MS-ceramide quantification methodology. The changes needed to adapt the LC-MS method mostly relate to the MS/MS method to include relevant ceramide species found in humans but not in porcine skin, such as the hydroxysphingosine-derived ceramides. Furthermore, the injection volume would have to be adjusted for the tape strips. Also, regarding the CRS system, options for measurements on human skin in vivo are available, which are associated with only a minor loss of data quality. The loss is due to the necessary reduction of the laser power, which must be reduced to avoid damaging the living skin due to intense, focused laser energy. Together, this method combination can facilitate an approach toward the development of nonirritating and, in the best case, barrier-restoring formulations. In the future, an in vivo study will support this ex-vivo approach and investigate the ex vivo–in vivo correlation.

## Supporting Information Available

The results of LC-MS measurements in detail for SEs and ceramides regarding ester composition of SEs, different ceramides by class and precursor, and product ion values(PDF)

## References

[ref1] Tfaili S.; Gobinet C.; Josse G.; Angiboust J. F.; Manfait M.; Piot O. Confocal Raman Microspectroscopy for Skin Characterization: A Comparative Study between Human Skin and Pig Skin. Analyst 2012, 137 (16), 3673–3682. 10.1039/C2AN16292J. 22754919

[ref2] Jacobi U.; Kaiser M.; Toll R.; Mangelsdorf S.; Audring H.; Otberg N.; Sterry W.; Lademann J. Porcine Ear Skin: An in Vitro Model for Human Skin. Skin Res. Technol. 2007, 13 (1), 19–24. 10.1111/j.1600-0846.2006.00179.x. 17250528

[ref3] Pasparakis M.; Haase I.; Nestle F. O. Mechanisms Regulating Skin Immunity and Inflammation. Nat. Rev. Immunol. 2014, 14, 289–301. 10.1038/nri3646. 24722477

[ref4] Bouwstra J. A.; Helder R. W. J.; El Ghalbzouri A. Human Skin Equivalents: Impaired Barrier Function in Relation to the Lipid and Protein Properties of the Stratum Corneum. Adv. Drug Delivery Rev. 2021, 175, 113802 10.1016/j.addr.2021.05.012. 34015420

[ref5] Herbig M. E.; Houdek P.; Gorissen S.; Zorn-Kruppa M.; Wladykowski E.; Volksdorf T.; Grzybowski S.; Kolios G.; Willers C.; Mallwitz H.; Moll I.; Brandner J. M. A Custom Tailored Model to Investigate Skin Penetration in Porcine Skin and Its Comparison with Human Skin. Eur. J. Pharm. Biopharm. 2015, 95, 99–109. 10.1016/j.ejpb.2015.03.030. 25857837

[ref6] Stella A.; Bonnier F.; Tfayli A.; Yvergnaux F.; Byrne H. J.; Chourpa I.; Munnier E.; Tauber C. Raman Mapping Coupled to Self-Modelling MCR-ALS Analysis to Estimate Active Cosmetic Ingredient Penetration Profile in Skin. J. Biophotonics 2020, 13 (11), e202000136 10.1002/jbio.202000136. 32678939

[ref7] Kourbaj G.; Gaiser A.; Bielfeldt S.; Lunter D. Assessment of Penetration and Permeation of Caffeine by Confocal Raman Spectroscopy in Vivo and Ex Vivo by Tape Stripping. Int. J. Cosmet Sci. 2023, 45 (1), 14–28. 10.1111/ics.12820. 36350131

[ref8] Krombholz R.; Fressle S.; Nikolić I.; Pantelić I.; Savić S.; Sakač M. C.; Lunter D. Ex Vivo–in Vivo Comparison of Drug Penetration Analysis by Confocal Raman Microspectroscopy and Tape Stripping. Exp. Dermatol. 2022, 31 (12), 1908–1919. 10.1111/exd.14672. 36055759

[ref9] Krombholz R.; Fressle S.; Lunter D. Ex Vivo-In Vivo Correlation of Retinol Stratum Corneum Penetration Studies by Confocal Raman Microspectroscopy and Tape Stripping. Int. J. Cosmet Sci. 2022, 44, 299–308. 10.1111/ics.12775. 35396727

[ref10] Vater C.; Apanovic A.; Riethmüller C.; Litschauer B.; Wolzt M.; Valenta C.; Klang V. Changes in Skin Barrier Function after Repeated Exposition to Phospholipid-Based Surfactants and Sodium Dodecyl Sulfate in Vivo and Corneocyte Surface Analysis by Atomic Force Microscopy. Pharmaceutics 2021, 13 (4), 436 10.3390/pharmaceutics13040436. 33804924 PMC8063842

[ref11] Liu Y.; Lunter D. J. Systematic Investigation of the Effect of Non-Ionic Emulsifiers on Skin by Confocal Raman Spectroscopy—a Comprehensive Lipid Analysis. Pharmaceutics 2020, 12 (3), 223 10.3390/pharmaceutics12030223. 32131544 PMC7150945

[ref12] Kendall A. C.; Kiezel-Tsugunova M.; Brownbridge L. C.; Harwood J. L.; Nicolaou A. Lipid Functions in Skin: Differential Effects of n-3 Polyunsaturated Fatty Acids on Cutaneous Ceramides, in a Human Skin Organ Culture Model. Biochim. Biophys. Acta, Biomembr. 2017, 1859 (9), 1679–1689. 10.1016/j.bbamem.2017.03.016. 28341437 PMC5504780

[ref13] Vavrova K.; Kovačik A.; Opalka L. Ceramides in the Skin Barrier. Eur. Pharm. J. 2017, 64 (2), 28–35. 10.1515/afpuc-2017-0004.

[ref14] Berkers T.; Visscher D.; Gooris G. S.; Bouwstra J. A. Topically Applied Ceramides Interact with the Stratum Corneum Lipid Matrix in Compromised Ex Vivo Skin. Pharm. Res. 2018, 35, 48 10.1007/s11095-017-2288-y. 29411158 PMC5801391

[ref15] Beddoes C. M.; Gooris G. S.; Barlow D. J.; Lawrence M. J.; Dalgliesh R. M.; Malfois M.; Demé B.; Bouwstra J. A. The Importance of Ceramide Headgroup for Lipid Localisation in Skin Lipid Models. BBA-Biomembranes 2022, 1864, 183886 10.1016/j.bbamem.2022.183886. 35143742

[ref16] Ge F.; Sun K.; Hu Z.; Dong X. Role of Omega-Hydroxy Ceramides in Epidermis: Biosynthesis, Barrier Integrity and Analyzing Method. Int. J. Mol. Sci. 2023, 24 (5), 5035 10.3390/ijms24055035. 36902463 PMC10003399

[ref17] Zhang Z.; Lunter D. J. Confocal Raman Microspectroscopy as an Alternative Method to Investigate the Extraction of Lipids from Stratum Corneum by Emulsifiers and Formulations. Eur. J. Pharm. Biopharm. 2018, 127, 61–71. 10.1016/j.ejpb.2018.02.006. 29428793

[ref18] Liu Y.; Ilić T.; Pantelic I.; Savić S.; Lunter D. J. Topically Applied Lipid-Containing Emulsions Based on PEGylated Emulsifiers: Formulation, Characterization, and Evaluation of Their Impact on Skin Properties Ex Vivo and in Vivo. Int. J. Pharm. 2022, 626, 122202 10.1016/j.ijpharm.2022.122202. 36122613

[ref19] Mojumdar E. H.; Kariman Z.; Van Kerckhove L.; Gooris G. S.; Bouwstra J. A. The Role of Ceramide Chain Length Distribution on the Barrier Properties of the Skin Lipid Membranes. Biochim. Biophys. Acta, Biomembr. 2014, 1838 (10), 2473–2483. 10.1016/j.bbamem.2014.05.023. 24875266

[ref20] Van Smeden J.; Boiten W. A.; Hankemeier T.; Rissmann R.; Bouwstra J. A.; Vreeken R. J. Combined LC/MS-Platform for Analysis of All Major Stratum Corneum Lipids, and the Profiling of Skin Substitutes. Biochim. Biophys. Acta, Mol. Cell Biol. Lipids 2014, 1841 (1), 70–79. 10.1016/j.bbalip.2013.10.002. 24120918

[ref21] Wertz P. W.; Downing D. T. Ceramides of Pig Epidermis: Structure Determination. J. Lipid Res. 1983, 24 (6), 759–765. 10.1016/S0022-2275(20)37950-5. 6886564

[ref22] Kawana M.; Miyamoto M.; Ohno Y.; Kihara A. Comparative Profiling and Comprehensive Quantification of Stratum Corneum Ceramides in Humans and Mice by LC/MS/MS. J. Lipid Res. 2020, 61 (6), 884–895. 10.1194/jlr.RA120000671. 32265320 PMC7269764

[ref23] Ilić T.; Savić S.; Pantelić I.; Marković B.; Savić S. Development of Suitable Working Protocol for in Vitro Tape Stripping: A Case Study with Biocompatible Aceclofenac-Loaded Topical Nanoemulsions. Symposium on Pharmaceutical Engineering Research SPhERe, Elsevier, 2019, 25, 27, 10.24355/dbbs.084-202001221146-0.

[ref24] Schoenfelder H.; Liu Y.; Lunter D. J. Systematic Investigation of Factors, Such as the Impact of Emulsifiers, Which Influence the Measurement of Skin Barrier Integrity by in-Vitro Trans-Epidermal Water Loss (TEWL). Int. J. Pharm. 2023, 638, 122930 10.1016/j.ijpharm.2023.122930. 37028576

[ref25] Tfayli A.; Guillard E.; Manfait M.; Baillet-Guffroy A. Raman Spectroscopy: Feasibility of in Vivo Survey of Stratum Corneum Lipids, Effect of Natural Aging. Eur. J. Dermatol. 2012, 22 (1), 36–41. 10.1684/ejd.2011.1507. 22075158

[ref26] Choe C.; Lademann J.; Darvin M. E. A Depth-Dependent Profile of the Lipid Conformation and Lateral Packing Order of the Stratum Corneum in Vivo Measured Using Raman Microscopy. Analyst 2016, 141 (6), 1981–1987. 10.1039/C5AN02373D. 26855232

[ref27] Zhang Z.; Lunter D. J. Confocal Raman Microspectroscopy as an Alternative to Differential Scanning Calorimetry to Detect the Impact of Emulsifiers and Formulations on Stratum Corneum Lipid Conformation. Eur. J. Pharm. Sci. 2018, 121, 1–8. 10.1016/j.ejps.2018.05.013. 29775655

[ref28] Karande P.; Jain A.; Arora A.; Ho M. J.; Mitragotri S. Synergistic Effects of Chemical Enhancers on Skin Permeability: A Case Study of Sodium Lauroylsarcosinate and Sorbitan Monolaurate. Eur. J. Pharm. Sci. 2007, 31 (1), 1–7. 10.1016/j.ejps.2007.01.004. 17368869

[ref29] Liu Y.; Binks B. P. Fabrication of Stable Oleofoams with Sorbitan Ester Surfactants. Langmuir 2022, 38 (48), 14779–14788. 10.1021/acs.langmuir.2c02413. 36410861 PMC9730906

[ref30] Fiume M. M.; Bergfeld W. F.; Belsito D. V.; Hill R. A.; Klaassen C. D.; Liebler D. C.; Marks J. G. Jr; Shank R. C.; Slaga T. J.; Snyder P. W.; Gill L. J.; Heldreth B. Safety Assessment of Sorbitan Esters as Used in Cosmetics. Int J Toxicol. 2019, 38 (2_suppl), 60S–80S. 10.1177/1091581819871877. 31522651

[ref31] Upadhyay K. K.; Tiwari C.; Khopade A. J.; Bohidar H. B.; Jain S. K. Sorbitan Ester Organogels for Transdermal Delivery of Sumatriptan. Drug Dev. Ind. Pharm. 2007, 33 (6), 617–625. 10.1080/03639040701199266. 17613026

[ref32] Lunter D. J. Determination of Skin Penetration Profiles by Confocal Raman Microspectroscopy: Statistical Evaluation of Optimal Microscope Configuration. J. Raman Spectrosc. 2017, 48 (2), 152–160. 10.1002/jrs.5001.

[ref33] Lunter D. J. How Confocal Is Confocal Raman Microspectroscopy on the Skin? Impact of Microscope Configuration and Sample Preparation on Penetration Depth Profiles. Skin Pharmacol. Physiol. 2016, 29 (2), 92–101. 10.1159/000444806. 27054960

[ref34] Liu Y.; Lunter D. J. Selective and Sensitive Spectral Signals on Confocal Raman Spectroscopy for Detection of Ex Vivo Skin Lipid Properties. Transl. Biophotonics 2020, 2, e202000003 10.1002/tbio.202000003.

[ref35] Kligman A. M. Preparation of Isolated Sheets of Human Stratum Corneum. Arch. Dermatol. 1963, 88 (6), 702 10.1001/archderm.1963.01590240026005. 14071437

[ref36] Liu Y.; Lunter D. J. Optimal Configuration of Confocal Raman Spectroscopy for Precisely Determining Stratum Corneum Thickness: Evaluation of the Effects of Polyoxyethylene Stearyl Ethers on Skin. Int. J. Pharm. 2021, 597, 120308 10.1016/j.ijpharm.2021.120308. 33540027

[ref37] Bridges T. E.; Houlne M. P.; Harris J. M. Spatially Resolved Analysis of Small Particles by Confocal Raman Microscopy: Depth Profiling and Optical Trapping. Anal. Chem. 2004, 76 (3), 576–584. 10.1021/ac034969s. 14750849

[ref38] Williams A. C.; Edwards H. G. M.; Barry B. W. Raman Spectra of Human Keratotic Biopolymers: Skin, Callus, Hair and Nail. J. Raman Spectrosc. 1994, 25, 95–98. 10.1002/jrs.1250250113.

[ref39] Snyder R. G.; Hsut S. L.; Krimm S. Vibrational Spectp in the C-H Stretching Region and the Structure of the Polymethylene Chain. Spectrochim. Acta Part A: Mol. Spectrosc. 1978, 34 (4), 395–406. 10.1016/0584-8539(78)80167-6.

[ref40] Stearate S. European Directorate for the Quality of Medicines & HealthCare. In European Pharmacopoeia (Ph. Eur.) 11th Edition; Council of Europe: Strasbourg, 2022.

[ref41] Sheskey P. J.; Hancock B. C.; Moss G. P.; Goldfarb D. J. Handbook of Pharmaceutical Excipients – Ninth Edition;Pharmaceutical Press: London, England, 2020.

[ref42] European Medicines Agency. Draft Guideline on Quality and Equivalence of Topical Products; 2018. www.ema.europa.eu/contact. accessed 24 October 2024.

[ref43] Törmä H.; Lindberg M.; Berne B. Skin Barrier Disruption by Sodium Lauryl Sulfate-Exposure Alters the Expressions of Involucrin, Transglutaminase 1, Profilaggrin, and Kallikreins during the Repair Phase in Human Skin in Vivo. J. Invest. Dermatol. 2008, 128 (5), 1212–1219. 10.1038/sj.jid.5701170. 18007579

[ref44] Liu Y.; Lunter D. J. Confocal Raman Spectroscopy at Different Laser Wavelengths in Analyzing Stratum Corneum and Skin Penetration Properties of Mixed PEGylated Emulsifier Systems. Int. J. Pharm. 2022, 616, 121561 10.1016/j.ijpharm.2022.121561. 35151816

[ref45] Liu Y.; Lunter D. J. Profiling Skin Penetration Using PEGylated Emulsifiers as Penetration Enhancers via Confocal Raman Spectroscopy and Fluorescence Spectroscopy. Eur. J. Pharm. Biopharm. 2021, 166, 1–9. 10.1016/j.ejpb.2021.04.027. 34082121

[ref46] Vater C.; Bosch L.; Mitter A.; Göls T.; Seiser S.; Heiss E.; Elbe-Bürger A.; Wirth M.; Valenta C.; Klang V. Lecithin-Based Nanoemulsions of Traditional Herbal Wound Healing Agents and Their Effect on Human Skin Cells. Eur. J. Pharm. Biopharm. 2022, 170, 1–9. 10.1016/j.ejpb.2021.11.004. 34798283

[ref47] Vater C.; Hlawaty V.; Werdenits P.; Cichoń M. A.; Klang V.; Elbe-Bürger A.; Wirth M.; Valenta C. Effects of Lecithin-Based Nanoemulsions on Skin: Short-Time Cytotoxicity MTT and BrdU Studies, Skin Penetration of Surfactants and Additives and the Delivery of Curcumin. Int. J. Pharm. 2020, 580, 119209 10.1016/j.ijpharm.2020.119209. 32165223

[ref48] Janssens M.; Van Smeden J.; Gooris G. S.; Bras W.; Portale G.; Caspers P. J.; Vreeken R. J.; Hankemeier T.; Kezic S.; Wolterbeek R.; Lavrijsen A. P.; Bouwstra J. A. Increase in Short-Chain Ceramides Correlates with an Altered Lipid Organization and Decreased Barrier Function in Atopic Eczema Patients. J. Lipid Res. 2012, 53 (12), 2755–2766. 10.1194/jlr.P030338. 23024286 PMC3494247

[ref49] Croda. Span 60 pharma; https://www.crodapharma.com/en-gb/product-finder/product/5545-span_1_60_1_pharma#product-brochures-and-guides. accessed 2 October 2024.

[ref50] United States Pharmacopeial Convention. The United States Pharmacopeia, The National Formulary; USP NF, United States Pharmacopeial Convention: 2019.

[ref51] Ohnari H.; Naru E.; Sakata O.; Obata Y. Distribution of Domains Formed by Lateral Packing of Intercellular in the Stratum Corneum. Chem. Pharm. Bull. 2023, 71 (1), 31–40. 10.1248/cpb.c22-00533. 36596510

[ref52] Van Smeden J.; Janssens M.; Gooris G. S.; Bouwstra J. A. The Important Role of Stratum Corneum Lipids for the Cutaneous Barrier Function ☆. BBA - Molecular And Cell Biology Of Lipids 2014, 1841, 295–313. 10.1016/j.bbalip.2013.11.006. 24252189

[ref53] Bouwstra J. A.; Ponec M. The Skin Barrier in Healthy and Diseased State. Biochim. Et Biophy. Acta - Biomembr. 2006, 1758, 2080–2095. 10.1016/j.bbamem.2006.06.021. 16945325

[ref54] Moghadam S. H.; Saliaj E.; Wettig S. D.; Dong C.; Ivanova M. V.; Huzil J. T.; Foldvari M. Effect of Chemical Permeation Enhancers on Stratum Corneum Barrier Lipid Organizational Structure and Interferon Alpha Permeability. Mol. Pharmaceutics 2013, 10 (6), 2248–2260. 10.1021/mp300441c. 23587061

[ref55] Takagi Y.; Nakagawa H.; Higuchi K.; Imokawa G. Characterization of Surfactant-Induced Skin Damage through Barrier Recovery Induced by Pseudoacylceramides. Dermatology 2005, 211 (2), 128–134. 10.1159/000086442. 16088159

[ref56] Imokawa G. Surfactant-Induced Depletion of Ceramides and Other Intercellular Lipids: Implication for the Mechanism Leading to Dehydration of the Stratum Corneum. Exogen. Dermatol. 2005, 3 (2), 81–98. 10.1159/000086158.

[ref57] Ananthapadmanabhan K. P.; Mukherjee S.; Chandar P. Stratum Corneum Fatty Acids: Their Critical Role in Preserving Barrier Integrity during Cleansing. Int. J. Cosmet Sci. 2013, 35, 337–345. 10.1111/ics.12042. 23363400

