# Supplementary material for: Ceramide Profiling of Porcine Skin and Systematic Investigation of the Impact of Sorbitan Esters (SEs) on the Barrier Function of the Skin
Source: Mol Pharm. 2025 Mar 11;22(4):2019–28. doi: 10.1021/acs.molpharmaceut.4c01245 (PMC11979889; doi:10.1021/acs.molpharmaceut.4c01245)
Supplement: Supplementary file 1 — mp4c01245_si_001.pdf [file mp4c01245_si_001.pdf]

Ceramides profiling of porcine skin and systematic investigation of the impact of sorbitan esters (SEs) on the barrier function of the skin

*Hans Schoenfelder1, Moritz Reuter1, Dirk-Heinrich Evers2, Michael E. Herbig2, Dominique Jasmin Lunter1**

1Department of Pharmaceutical Technology, Faculty of Science, Eberhard Karls Universität Tübingen, Auf der Morgenstelle 8, 72076 Tuebingen, Germany

2RaDes GmbH, Schnackenburgallee 114, 22525, Hamburg, Germany

Supporting information.

Content:

Table S1

Table S2

Table S3

The information shows the results of LC-MS measurements in detail for SEs and ceramides.

Table S1. Supporting material: Overview of experimental and calculated saponification values of different SE variants.

Emulsifier name	batch	Nominal composition	SV calculated for 100% sorbitan monoesters	Specification for SV	SV measured (CoA)	SV calculated from peak area distribution (mono/di/tri)	% of measured SV

SE 40	#0001767384	sorbitan monopalmitate	139.4	140 - 155	148.0	173.1	117.0
SE 60	#0001616251	sorbitan monostearate	134.6	147 - 157	150.0	165.2	110.2
SE 80	#0001959128	sorbitan monooleate	131.4	145 - 160	155.0	160.3	103.4
SE 120	#0001949925	sorbitan monoisostearate	131.0	140 - 158	151.0	157.3	104.2
						Mean:	108.7
						%RSD:	5.8

Table S2. Relative abundances of ceramide species of the porcine SC. Relative abundance in the case of the ceramide chain lengths is calculated for ω-esterified ceramides and regular ceramides separately, as the measured range of ceramide chain length of the two groups differ. Asterisks denote chain lengths belonging to ω-esterified ceramides. Sphingosine- and dihydrosphingosine-based ceramide classes as well as chain lengths of 16, 24 and 26 carbon atoms dominate the picture for the regular ceramide classes. For ω-esterified ceramides, the most abundant ceramides are of the ceramide class EOS and contain 30 carbon atoms in their fatty acid chain. Values are given in percent ± SD.

Ceramide Class:	NS	NDS	AS	ADS	NP	AP	EODS	EOS	EOP												
Relative Abundance in Percent by Weight:	37.92 ± 8.57	38.08 ± 2.63	8.54 ± 0.58	4.20 ± 0.53	5.99 ± 0.81	4.09 ± 0.59	0.06 ± 0.02	1.11 ± 0.28	0.01 ± 0.00												
Ceramide Chain Length (Number of Carbon Atoms):	16	17	18	19	20	21	22	23	24	25	26	27	28	28*	29*	30*	31*	32*	33*	34*	35*

	32.61 ± 3.01	1.15 ± 0.16	3.82 ± 0.18	0.95 ± 0.05	12.18 ± 1.79	1.35 ± 0.22	8.91 ± 0.29	2.62 ± 0.43	18.46 ± 1.72	2.45 ± 0.34	8.91 ± 1.00	1.07 ± 0.16	5.52 ± 0.50	13.42 ± 0.66	6.07 ± 2.53	52.99 ± 14.26	6.20 ± 2.50	16.96 ± 4.92	1.25 ± 0.43	2.94 ± 0.78	0.17 ± 0.05

Table S3. Overview of ceramides by precursor ion, product ion, collision energy, and retention time.

Ceramide Species Name	Precursor m/z	Product m/z	Collision Energy (volts)	Retention Time (Min)
NS16	538.51	264.4	-29	8.86
NDS16	540.5	284.4	-29	9.05
NS17	552.53	264.4	-29	9.16
NDS17	554.5	284.4	-29	9.35
NS18	566.54	264.4	-29	9.43
NDS18	568.6	284.4	-29	9.63
NDS19	582.6	284.4	-29	9.9
NS19	580.56	264.4	-29	9.72
NS20	594.17	264.4	-30	10
NS21	608.19	264.4	-30	10.28
NS22	622.41	264.4	-30	10.55
NS23	636.22	264.4	-30	10.85
NS24	650.34	264.4	-30	11.13
NS25	664.25	264.4	-30	11.42
NS26	678.37	264.4	-30	11.72
NS27	692.38	264.4	-30	12.03
NS28	706.4	264.4	-30	12.32

NDS20	596.6	284.4	-25	10.2
NDS21	610.6	284.4	-25	10.46
NDS22	624.6	284.4	-25	10.74
NDS23	638.6	284.4	-25	11.03
NDS24	652.6	284.4	-25	11.33
NDS25	666.6	284.4	-25	11.63
NDS26	680.7	284.4	-25	11.92
NDS27	694.7	284.4	-25	12.22
NDS28	708.7	284.4	-25	12.53
NS16:1	518.5	264.4	-25	8.64
NS24:1	630.6	264.4	-25	10.56
NS26:1	658.7	264.4	-25	11.13
NDS16:1	538.51	284.4	-29	8.93
NDS24:1	650.34	284.4	-30	10.91
NDS26:1	678.37	284.4	-30	11.35
EODS28	987	284.4	-49	13.77
EODS29	1001	284.4	-49	13.9
EODS30:1	1012.97	284.4	-49	13.77
EODS32:1	1041	284.4	-49	14
EODS30	1014.97	284.4	-49	14
EODS31	1028.99	284.4	-49	13.95
EODS32	1043	284.4	-49	14.04

EODS32:1	1041	284.4	-49	13.88
EODS33	1057	284.4	-49	14.1
EODS34	1071.1	284.4	-49	14.33
EODS34:1	1069.1	284.4	-49	13.74
EODS35	1085.1	284.4	-49	14.23
EOS28	984.94	264.4	-49	13.77
EOS29	998.96	264.4	-49	13.87
EOS30	1012.97	264.4	-49	13.96
EOS31	1026.99	264.4	-49	14.05
EOS32	1041	264.4	-49	14.13
EOS33	1055.02	264.4	-49	14.21
EOS34	1069.03	264.4	-49	14.29
EOS35	1083.05	264.4	-49	14.38
EOS30:1	1011	264.4	-49	13.77
EOS32:1	1039	264.4	-49	13.95
EOS34:1	1067.1	264.4	-49	14.12
AP16	572.6	300.4	-27	8.35
AP16:1	570.6	300.4	-27	7.64
AP17	586.6	300.4	-27	8.61
AP18	600.6	300.4	-27	8.88
AP19	614.6	300.4	-27	9.17
AP20	628.6	300.4	-33	9.44

AP21	642.6	300.4	-33	9.73
AP22	656.7	300.4	-33	10.06
AP23	670.7	300.4	-33	10.31
AP24	684.7	300.4	-33	10.62
AP25	698.7	300.4	-33	10.89
AP26	712.7	300.4	-33	11.18
AP24:1	682.7	300.4	-33	10.04
AP26:1	710.7	300.4	-33	10.59
AP27	726.7	300.4	-33	11.48
AP28	740.8	300.4	-33	11.78
NP16	556.6	300.4	-28	8.56
NP16:1	554.6	300.4	-28	7.76
NP17	570.6	300.4	-28	8.86
NP18	584.6	300.4	-28	9.12
NP19	598.6	300.4	-28	9.4
NP20	612.6	300.4	-31	9.68
NP21	626.6	300.4	-31	9.98
NP22	640.7	300.4	-31	10.26
NP23	654.7	300.4	-31	10.55
NP24	668.7	300.4	-31	10.84
NP25	682.7	300.4	-31	11.14
NP26	696.7	300.4	-31	11.44

NP24:1	666.7	300.4	-31	10.28
NP26:1	694.7	300.4	-31	10.85
NP27	710.7	300.4	-31	11.72
NP28	724.8	300.4	-31	12.05
EOP28	1003	300.4	-49	13.67
EOP29	1017	300.4	-49	13.78
EOP30	1031	300.4	-49	13.88
EOP30:1	1029	300.4	-49	13.67
EOP31	1045	300.4	-49	13.96
EOP32	1059	300.4	-49	14.05
EOP32:1	1057	300.4	-49	13.87
EOP34	1087.75	300.4	-49	14.38
EOP34:1	1085.1	300.4	-49	14.2
EOP33	1073	300.4	-49	14.3
EOP35	1101.1	300.4	-49	14.51
AS16	554.51	264.4	-28	8.67
AS17	568.52	264.4	-28	8.94
AS18	582.54	264.4	-28	9.2
AS19	596.55	264.4	-28	9.47
AS20	610.27	264.4	-32	9.75
AS21	624.49	264.4	-32	10.03
AS22	638.3	264.4	-32	10.31

AS23	652.22	264.4	-32	10.59
AS24	666.23	264.4	-32	10.83
AS25	680.35	264.4	-32	11.15
AS26	694.36	264.4	-32	11.44
AS27	708.38	264.4	-32	11.74
AS28	722.39	264.4	-32	12.03
AS16:1	552.5	264.4	-28	7.96
AS24:1	664.6	264.4	-32	10.31
AS26:1	692.7	264.4	-32	10.85
ADS16	556.5	284.4	-29	8.8
ADS17	570.5	284.4	-30	9.17
ADS18	584.6	284.4	-30	9.34
ADS19	598.6	284.4	-30	9.62
ADS20	612.6	284.4	-35	9.88
ADS21	626.6	284.4	-35	10.18
ADS22	640.6	284.4	-35	10.47
ADS23	654.6	284.4	-35	10.76
ADS24	668.6	284.4	-35	11.05
ADS25	682.6	284.4	-35	11.36
ADS26	696.7	284.4	-35	11.66
ADS27	710.7	284.4	-35	11.96
ADS28	724.7	284.4	-35	12.28

ADS16:1	554.51	284.4	-28	8.59
ADS24:1	666.23	284.4	-32	10.49
ADS26:1	694.36	284.4	-32	11.02
